# Working memory is supported by learning to represent items as actions

**DOI:** 10.3758/s13414-023-02654-z

**Published:** 2023-03-01

**Authors:** Aaron Cochrane, C. Shawn Green

**Affiliations:** 1 University of Geneva, Geneva, Switzerland; 2 University of Wisconsin-Madison, Madison, Wisconsin USA

**Keywords:** Memory: Visual working and short-term memory, Perception and Action, Attention in learning

## Abstract

Working memory is typically described as a set of processes that allow for the maintenance and manipulation of information for proximal actions, yet the “action” portion of this construct is commonly overlooked. In contrast, neuroscience-informed theories of working memory have emphasized the hierarchical nature of memory representations, including both goals and sensory representations. These two representational domains are combined for the service of actions. Here, we tested whether, as it is commonly measured (i.e., with computer-based stimuli and button-based responses), working memory involved the planning of motor actions (i.e., specific button presses). Next, we examined the role of motor plan learning in successful working memory performance. Results showed that visual working memory performance was disrupted by unpredictable motor mappings, indicating a role for motor planning in working memory. Further, predictable motor mappings were in fact learned over the course of the experiment, thereby causing the measure of working memory to be partially a measure of participants’ ability to learn arbitrary associations between visual stimuli and motor responses. Such learning was not highly specific to certain mappings; in sequences of short tasks, participants improved in their abilities to learn to represent items as actions in working memory. We discuss implications for working memory theories in light of hierarchical structure learning and ecological validity.

**Supplementary Information:**

The online version contains supplementary material available at 10.3758/s13414-023-02654-z.

## Introduction

Real-world situations that demand the use of working memory abound, from hearing a telephone number and then typing it a few moments later, to reading a recipe and then walking throughout the kitchen to gather the necessary ingredients, to looking at a figure in the assembly instructions for a piece of furniture and then performing the described steps in the correct order. These situations often require a combination of novel task demands and familiar ones, with some degree of generalization possible from previous knowledge to the present behaviors.

Critically, while the examples above each demonstrate the *action-oriented* nature of working memory (i.e., that the everyday utilization of working memory relies on goal context and the actions necessary to reach goals), conventional perspectives in the field have focused on more abstract *information-level* considerations (e.g., as related to the capacity of working memory or the nature of representations in working memory; Fukuda et al., 2010; Ma et al., 2014; Shipstead et al., 2014; van den Berg et al., 2014; Vogel et al., 2001). While debates on such information-level topics are extremely important for characterizing the domain of working memory, it is notable that the use-oriented and goal-directed aspects of working memory have been considered much less than the more abstract information-level considerations.

Indeed, the tasks commonly used to investigate working memory arguably reflect at least an implicit, if not an explicitly recognized, desire to measure the construct independently of the influence of motor action processes. While almost all tasks necessarily demand some sort of motor response, the required actions in most previous work have been predictable (e.g., using natural or well-learned mappings between each possible stimulus or decision and the associated action) or even knowable without any prior task-specific knowledge (e.g., using a mouse to click on a sequence of stimuli or using automatically verbalizable labels such as numbers or letters). Using tasks with minimal action-associated load is sensible given a strong emphasis only on information-level considerations. Yet, if real-world uses of working memory occur in the contexts of actions, then the failure to consider the role of motor processes may provide an incomplete account of working memory from an ecological perspective. For example, contemporary theories of the neuroscience of working memory highlight the abilities of people to flexibly attend to stable memory representations (e.g., long-term semantic memories) as a fundamental mechanism of working memory maintenance and retrieval (Engle, 2002) and stable representations have been considered in, for example, basic visual features or in explicit verbalizable knowledge (D’Esposito & Postle, 2015). Yet, when completing real-world actions such as cooking a recipe, the relevant information may not be maintained (at least not purely) in terms of attention to abstract verbal information, but rather in terms of the actions necessary (beat the eggs, zest the lemon, turn on the oven). In such a set of actions to complete, attention to a purely verbal coding would constrain the cook to using a highly impoverished set of information. Instead, to the extent that the cook knows how to zest a lemon, it seems likely that the memory would be stored in terms of the action necessary.

### Working memory as the representation of future goal-relevant actions

While clearly less common than work starting from a more abstract-information perspective, there is a growing body of evidence for the role of goal-oriented actions in working memory representations (van Ede & Nobre, 2022). For example, evidence for a common representation for verbal working memory and verbal production was drawn from similar patterns of errors in “tongue twister” sets of verbal information when compared with stimulus sets that were structured to be phonologically easier to parse (Acheson & MacDonald, 2009). Follow-up studies using transcranial magnetic stimulation (TMS) demonstrated that perturbation of similar neural circuits (i.e., posterior superior temporal gyrus) appeared to be selectively disruptive of both a verbal reading task and a verbal working memory task, but not a picture naming task, further supporting the proposition that common representations are necessary for action and maintenance of verbal working memory (Acheson et al., 2011).

Complementary results have been observed in the visuospatial domain. Whereas the verbal domain’s production system is itself verbal, the visuospatial production system is likely to be spatial in naturalistic contexts (e.g., motor movement of fingers or legs). Exploiting this fact, it has been observed that ambiguity in the motor responses required to successfully complete a task (i.e., pronation vs supination of the hand and wrist to match a remembered target angle) allows that response to be biased by the presentation of other presented stimuli that, while not explicitly relevant to the subsequent motor response, nonetheless bias the probability of supination or pronation on a given trial (Gallivan et al., 2016). The authors found that the motor demands of the nontarget stimuli essentially bled into the motor execution of the target stimuli, thereby providing evidence that the memory of the target itself was at least partially represented in terms of its motor execution.

More direct evidence for the common representation of visuospatial and motor information in working memory has come from observing the similar time courses of motor and visual EEG activity during a working memory task involving lateralized responses (i.e., using the left or right hand; van Ede et al., 2019). Neural activity was much more indicative of parallel activation rather than sequential activation, the latter of which would be expected if (as traditional theories of WM tend to tacitly assume) visuospatial working memory were to be stored as visual and spatial (i.e., non-motor) representations. Indeed, response-related aspects of working memory performance may at first glance appear to be restricted to “retrieval” processes, but the parallel activation observed in this study provides evidence for working memory maintenance using action codes as well.

The extant work has thus provided compelling evidence for the maintenance of working memory representations in action-planning codes (Olivers & Roelfsema, 2020; Trentin et al., 2023; van Ede & Nobre, 2022). These advances have brought experimental work on working memory progressively closer to the putative ecological applications of working memory. Nonetheless, like simple two-alternative forced-choice tasks or block-tapping tasks, previous studies on the action-oriented nature of working memory have not addressed the fact that *attention to action plans* is likely to dynamically interact in real-world situations with *learning of action plans.* That is, the stable representations utilized during working memory maintenance must themselves frequently be learned. These dynamic interactions between working memory and learning have yet to be thoroughly explored (cf. Kruijne et al., 2021; Zambrano et al., 2021), which motivated us to investigate whether learning action plans supports working memory performance.

### Working memory as the product of learning the actions relevant to a goal context

Models of prefrontal cortex activity in working memory performance have supported the proposition that initially unorganized neural activity may rapidly become structured in response to task constraints and informative feedback (Savin & Triesch, 2014). That is, in order to successfully deploy working memory for a particular task, a certain amount of task learning must be achieved. Such a perspective parallels that linking language production and verbal working memory; in order to represent verbal information in terms of a production representation, extensive language-specific learning is likely to be necessary (Acheson & MacDonald, 2009). In day-to-day situations these learning processes are likely to be characterized, not by long trajectories of learning, but instead by the rapid use of current goals and task constraints to determine what features are relevant for attention and maintenance in memory. In this sense, the act of effectively and flexibly deploying working memory is itself reliant on processes such as reinforcement learning that operate on somewhat longer (but still fairly short) timescales.

Such propositions are compatible with theories that emphasize the nature of working memory as utilizing the goal-oriented activation of long-term memory representations (Cowan, 1995; D’Esposito & Postle, 2015). Yet, despite the likely ecological utility of on-the-fly learning of relevant memory representations, the extent to which on-the-fly stable representations emerge that may subsequently be dynamically used by working memory remains to be explored. Given the hierarchically structured nature of information and tasks in the real world, it seems likely that a combination of novel learning and generalization from previous experiences is necessary (Botvinick, 2012; Kruijne et al., 2021).

### Does rapid learning play a role in working memory tasks that have typically disregarded a role for learning?

Despite the wealth of evidence indicating the possibility of an important role for learning task-relevant actions for the effective use of working memory, such learning is only rarely considered directly. This state of affairs is often by design and by convention; cognitive psychology has typically attempted to avoid “learning effects” or “order effects” through various methodological choices. For instance, the use of relatively short tasks is partially justified by the idea that longer tasks may be more sensitive to learning. Alternatively, when longer tasks are implemented, often participants must complete some number of “practice” trials prior to the “real” working memory task. Here, it is worth noting that while both approaches are quite common (Foster et al., 2015; Jaeggi et al., 2010; Unsworth & Engle, 2007; van den Berg et al., 2012), they actually make opposite assumptions. The former approach assumes that total naïveté leads to the purest measurement of the construct of interest, while the latter approach assumes that allowing for practice improves the subsequent measurement of the construct. Each of these perspectives has value. Unfortunately, methodological decisions often lead to their mutual incompatibility. In contrast, when using time-sensitive measurements of participants’ trajectories of performance over a working memory task, each of the perspectives is vindicated using a unified methodological and analytical framework. That is, by modeling multiple components of performance trajectories, even tasks as short as 64 trials can allow for dissociable effects of early-trial dynamic changes and late-trial stabilized performance measures (Cochrane & Green, 2021).

Previous work on trajectories of performance in working memory tasks have shown the utility of considering participants’ learning, for instance, by highlighting multiple dissociable links between working memory and fluid intelligence task performance (Cochrane & Green, 2021). While this work showed that learning occurs even over a somewhat small number of working memory task trials, and that considering the learning trajectory allowed for deeper inferences regarding links between working memory and intelligence task performance, this previous work could not address *what* was being learned. In the majority of canonical working memory measures it is evident that many dimensions of the task environment could be learned and lead to improved performance. These dimensions range from the identities of possible stimuli (e.g., images, sounds, colors, shapes), to temporal structure (i.e., at what cadence will stimuli appear), to the motor actions necessary (e.g., turning a knob, physically tapping on a series of items, pressing one of two buttons). Many other dimensions are more subtle but potentially may assist learning. Previous work has shown that sequences of tasks sharing structural features, such as stimulus timing, may lead to accelerated learning in novel tasks even when the superficial features between tasks are different enough that no immediate generalization is observed (Kattner, Cochrane, Cox, et al., 2017a). Modeling of working memory tasks using reinforcement learning approaches has extended learning-to-learn into the realm of working memory proper (Kruijne et al., 2021), yet behavioral evidence for such effects has been scarce.

Given the many reasons to believe that real-world deployment of working memory includes representations of learned goal-oriented actions, we thus sought to test whether those goal-oriented actions are measurably learned on the timescale of a typical working memory task. That is, we used action representations as a clear and ecologically relevant feature of working memory that can be learned on short timescales.

### The current experiments: WM in which action plans are learnable versus nonlearnable

Here we test whether, as it is commonly measured (i.e., with computer-based stimuli and button-based responses), putatively visual working memory involves the planning of motor actions (i.e., specific button presses). Such a demonstration allows for the investigation of action planning in working memory without the use of specialized hardware (e.g., to measure EEG or reaching movements; cf. pressing one of two buttons until a probe stimulus matches a target; Boettcher et al., 2021). Next, we examine the role of *learning to make action plans* in successful working memory performance. Then, because condition differences may be due to recall disruption, we test a learnable action-planning condition in which we implement a secondary search task interrupting recall. That is, WM has been often studied by identifying disrupting factors; if a secondary task disrupts WM performance, shared cognitive load presumably exists. We use such a disrupting secondary task to demonstrate that, despite overall decreases in performance, learning action mappings still occurs.

Next, in Experiment 2, we address two ways in which Experiment 1 lacked both ecological validity and links to previous experiments in working memory. Both real-world behaviors and behavioral research tasks are often supported by on-the-fly learning on reasonably short timescales. Given this, in Experiment 2 we examined learning within single blocks of 30 trials each. Further, since a crucial ability in the real world (and an underaddressed phenomenon in behavioral research) is the generalization of learning, in Experiment 2 we test whether within-block consistency in stimulus–response mappings helps improve learning to represent working memory memoranda. Evidence for this phenomena would indicate learning-to-learn, or hierarchical learning (Henin et al., 2021; Kattner, Cochrane, Cox, et al., 2017).

## General methods

### Sample characteristics

Undergraduates enrolled in an Introduction to Psychology course were provided extra credit in return for completing one of the five conditions from the two experiments described below. All participants read and signed a consent form. All procedures were approved by the University of Wisconsin ethics board. The target sample size was 30 participants in each of the five conditions, and a sample of 151 (81 female; age mean = 19.2, *SD* = 0.99; 71% White, 25% Asian, 3% multiple races, 1% other) participants are reported here (see Supplementary Information Table S1). An additional 29 participants were excluded for failing to have a capacity of at least one item, when calculating capacity across all trials (capacity calculated with a Weibull psychometric function maximum-likelihood fit to each participant’s data; 50% accuracy threshold divided by 2).

### Procedure

**Fig. 1:**
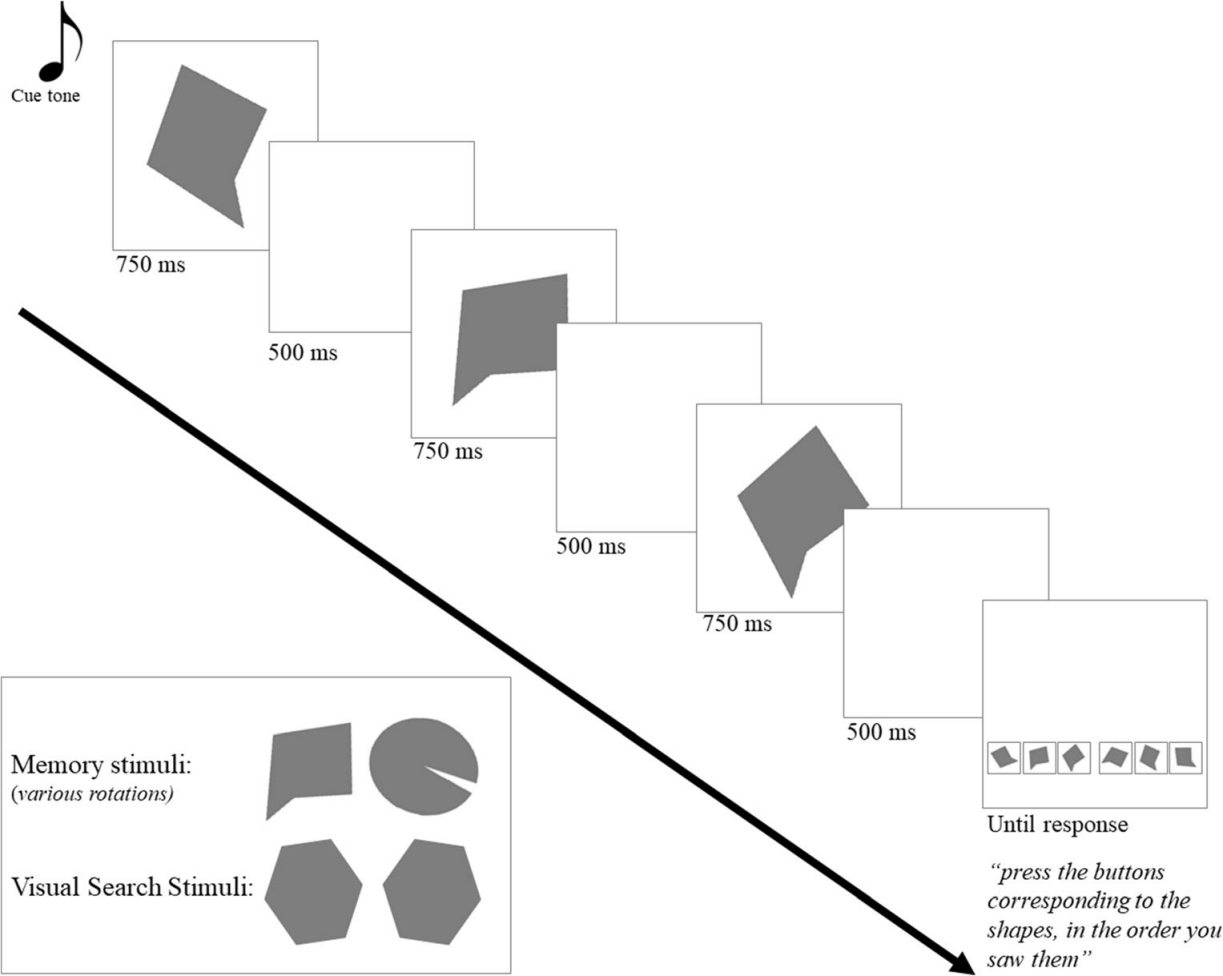
Span task used in the experiment. Set Size 3, with no visual search task, is shown as an example. In the conditions including the secondary visual search task, the search display was presented immediately preceding the memory response screen

All participants across both experiments (see below) completed four blocks of a visual working memory span task. Each block consisted of 30 trials, with memory span Set Sizes 2 through 6 presented in a partially randomized order. The first five trials of each block were in the order 2 3 4 5 6 to retain consistency; after that, presentation was fully randomized. Stimuli on two of the blocks (either first and third or second and fourth) were an oval with a wedge removed, while stimuli on the remaining blocks were an irregular pentagon (see Fig. 1). Within each block, distinctive stimuli were generated by rotating the block’s stimulus to six unique configurations 28 degrees apart. Rotations across blocks (e.g., the stimulus sets in Blocks 1 and 3) used nonoverlapping sets of angles (e.g., Block 1 may have been mostly pointing “upward” while Block 3 may have been pointing “downward”). That is, relative to a rotation of zero (e.g., the oval’s wedge pointing directly left), one block with the oval would have rotations of [20 48 76 104 132 160] while the other block with the oval would have rotations of [200 228 256 284 312 340]. Each block therefore had separate stimulus sets that were visually discriminable and fell along a counterclockwise-to-clockwise dimension.

The critical manipulation in this task was the mappings between stimuli and responses. Specific stimuli (e.g., the pentagon rotated at 76 degrees) could in principle be mapped to any arbitrary response. Here, the responses were mapped to six buttons, three for each hand, indicated by blank covered keys on a regular keyboard. Across the five between-subjects conditions in the two experiments, the response predictability was manipulated without any explicit instructions to the participants about response mappings (two conditions in Experiment 1 and two conditions in Experiment 2; see below). Given our interest in assessing the extent that learning of actions plays a role in the measurement of working memory task performance, both experiments used a shared “random-mappings” control condition that made motor learning impossible. In the “random mappings” condition, each stimulus was randomly assigned to a response button on every trial. The predictability of the necessary motor response was thus removed. In the absence of the ability to encode or maintain actions, the task became a test of only visual memory (i.e., encoding and maintenance of visual stimuli) rather than allowing any motor action planning during the stimulus presentation and delay periods. The data from these random-mapping participants was used as a comparison condition for each experiment.

### Analytical methods

Traditional approaches to examining learning typically divide behavior into blocks of trials which are then compared. However, in many learning contexts, within-block changes in performance are likely to be present. In such situations modeling the time-dependent improvements as a continuous parametric function of trial number presents various benefits for understanding the detailed time-course of change (Cochrane & Green, 2021; Kattner, Cochrane, & Green, 2017b; Zhang et al., 2019). In the present studies, time-evolving models used the *TEbrm* function from the R package *TEfits* (Cochrane, 2020), which used Bayesian modeling in Stan using the *brms* package (Bürkner, 2017). In short, trial-wise accuracy was modeled as arising from a Weibull [Quick] psychometric function with 100% accuracy on a set size of zero and a 16.67% accuracy with an arbitrarily large set size (i.e., a guessing rate of 1/6; Cochrane & Green, 2021; Wichmann & Hill, 2001). This psychometric function has two by-participant parameters, a constant shape and a threshold that may vary due to many factors (e.g., learning, task difficulty). The threshold represents the memory span set size at which a certain level of accuracy is reached; here, we chose a 50% threshold to retain some heuristic interpretability as “twice the number of items successfully recalled from WM.” For example, if a participant’s accuracy was 50% at a set size of four (i.e., threshold of four), then that would indicate that they could respond correctly for two items (noting that this is a heuristic, not mechanistic, interpretation). Thresholds and time constants of change were estimated on log scales. Fully disaggregated data was modeled, with each stimulus within each trial being associated with either a binary correct or an incorrect response for that stimulus at that point in the within-trial sequence. For model code, including formulas of psychometric functions and nonlinear learning models, see the Supplementary Information. Parameter reliability was determined using the posterior distributions of parameters or the differences between parameters’ posterior distributions (i.e., 95% CI falling above or below zero; see also Supplemental Figs. S3–S6 for comparisons with aggregated raw data).

## Experiment 1: The role of learning actions in visual working memory

### Experiment 1a: Does visual working memory involve learning motor action representations?

In Experiment 1a, performance in two conditions were contrasted. The first was a condition that closely followed typical cognitive psychology experiments (“constant mappings”), where the mappings of stimuli to responses was constant across blocks (i.e., within each block, the most-counterclockwise stimulus was always mapped to the left hand’s middle response, while the most-clockwise stimulus was always mapped to the right hand’s left response, etc.). All participants in this constant-mappings condition utilized the same stimulus–response mapping. The second condition, as noted above, utilized random mappings.

As described briefly above, threshold was modeled as changing as an exponential function of overall trial number (i.e., 1 to 120; see also Supplementary Information). By-participant random intercepts for the three parameters of the exponential were also included (i.e., the starting threshold, amount of time taken to 50% of change, and asymptotic threshold). In all cases we expected learning to be evidenced by within-condition starting thresholds being lower than the corresponding asymptotic thresholds. Further, we expected learning to be apparent in the form of higher asymptotic thresholds for the learnable-mappings conditions when compared with the random-mappings conditions (note that this same prediction holds for Experiment 1b). Differences in the amount of time taken to change may indicate accelerated learning in certain situations, but this parameter is difficult to interpret in the absence of reliably different starting and asymptotic thresholds.

#### Results

As a first test of whether working memory performance was worse when participants were unable to encode and maintain information in terms of consistent motor mappings, we fit generalized linear mixed-effects models using the R package *lme4* (Bates et al., 2015). When comparing accuracy on the last block of the task between the constant-mappings condition (*n* = 31) and the random-mapping condition (n = 30), the random-mapping condition was associated with reliably lower accuracy (*b* = −0.561, CI_95_ = [−0.987, −0.134]; the GLMEM used a binomial family and included fixed effects of condition and stimulus type, as well as by-participant random effects and by-participant set-size random slopes). When using a similar model using all 120 trials, however, such a reliable difference was not evident (*b* = −0.207, CI_95_ = [−0.474, 0.06]). This discrepancy indicated that the condition differences were likely to be due to learning of the motor mappings, and as such, our subsequent analyses utilize models that are sensitive to the detailed time courses of change over the course of the task.

**Fig. 2:**
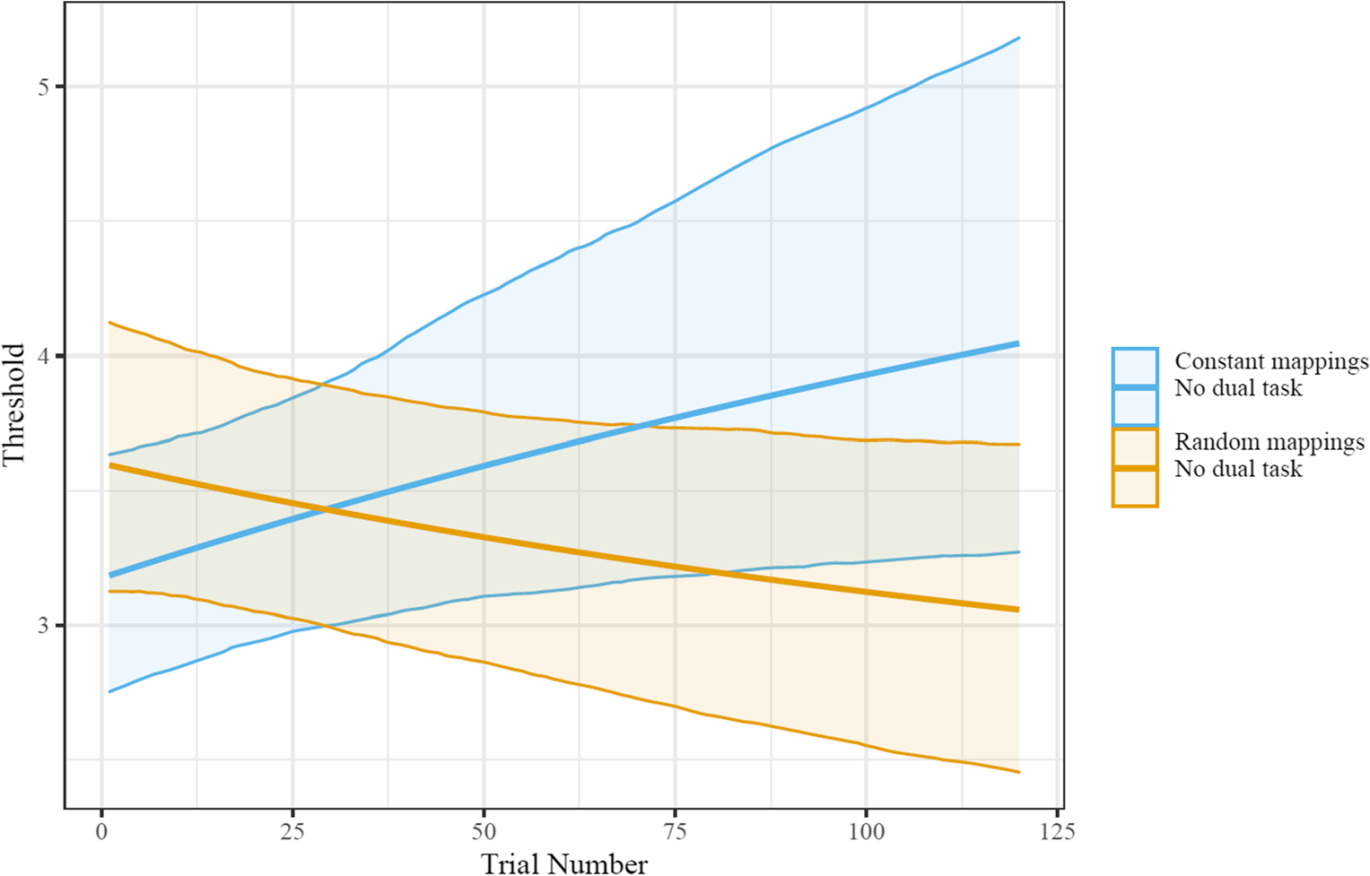
Group-level fits of change in thresholds (i.e., set size associated with 50% accuracy). The constant-mappings no-visual-search condition showed learning, while the random-mappings condition did not. Shaded area indicates the 95% CI of model fit values for each set size. For a visualization of the change in psychometric function as well as set sizes' accuracies over time, see the Supplementary Information (Figs. S1, S3, S5)

The Bayesian generalized nonlinear mixed-effects model had satisfactory convergence indices (i.e., all r-hats below 1.02 and all tail effective sample sizes above 500). When modeling the 50% accuracy threshold’s change as an exponential change of overall trial number, we found that the constant-mappings condition showed reliable improvements [increases] in threshold (*b* = 0.583, CI_95_ = [0.075,1.139]; see Figs. 2 & S3). Further, there was no reliable initial difference between the constant-mappings condition and the random-mappings condition (*b* = 0.122 CI_95_ = [−0.063,0.308]). In stark contrast, by the end of the 120 trials, the random-mapping condition showed reliably lower thresholds than the constant-mappings condition (*b* = −0.907 CI_95_ = [−1.749, −0.233]). These two results provided evidence that the constant-mappings condition achieved superior performance due to learning of their stimulus–response mappings. When specifically testing for whether participants in the random-mapping condition learned, there was not a reliable difference between asymptotic thresholds and starting thresholds (*b* = −0.430, CI_95_ = [−1.064, 0.057] ; see Supplemental Information Fig. S3).

#### Discussion

Participants’ visual working memory threshold improved over the course of 120 trials when stimulus–response mappings were learnable, but not when they were random. This provides some evidence for a role within working memory for on-the-fly learning to represent memoranda as motor actions. However, there remains a question regarding whether the differences between the above two conditions could be attributable to an extra task demand (i.e., in the random-mapping condition, participants had to visually search for target locations prior to responding).

### Experiment 1b: Are the condition differences in Experiment 1a due to disrupted recall (e.g., visual search)?

To test for the possibility that the results in Study 1a were simply due to the introduction of an intervening search task and not due to working memory directly interacting with action plans, we implemented a constant-mappings condition with an intervening visual search task. This condition was identical to the above constant-mappings no-visual-search constant-mappings condition with one exception: After stimulus presentation and prior to response availability, there was a simple visual search task. Six hexagons were presented on the screen in the same locations that the targets would subsequently be presented (see Fig. 1). Five of the hexagons were tilted slightly to one direction while the sixth, in a random location, was tilted slightly to the other direction. Participants were instructed to find the oddball shape and say aloud “left” or “right” to indicate the side with the oddball. This verbalization then triggered the experiment to continue to the span recall portion of the trial. The verbal answers were not recorded.

#### Results

**Fig. 3:**
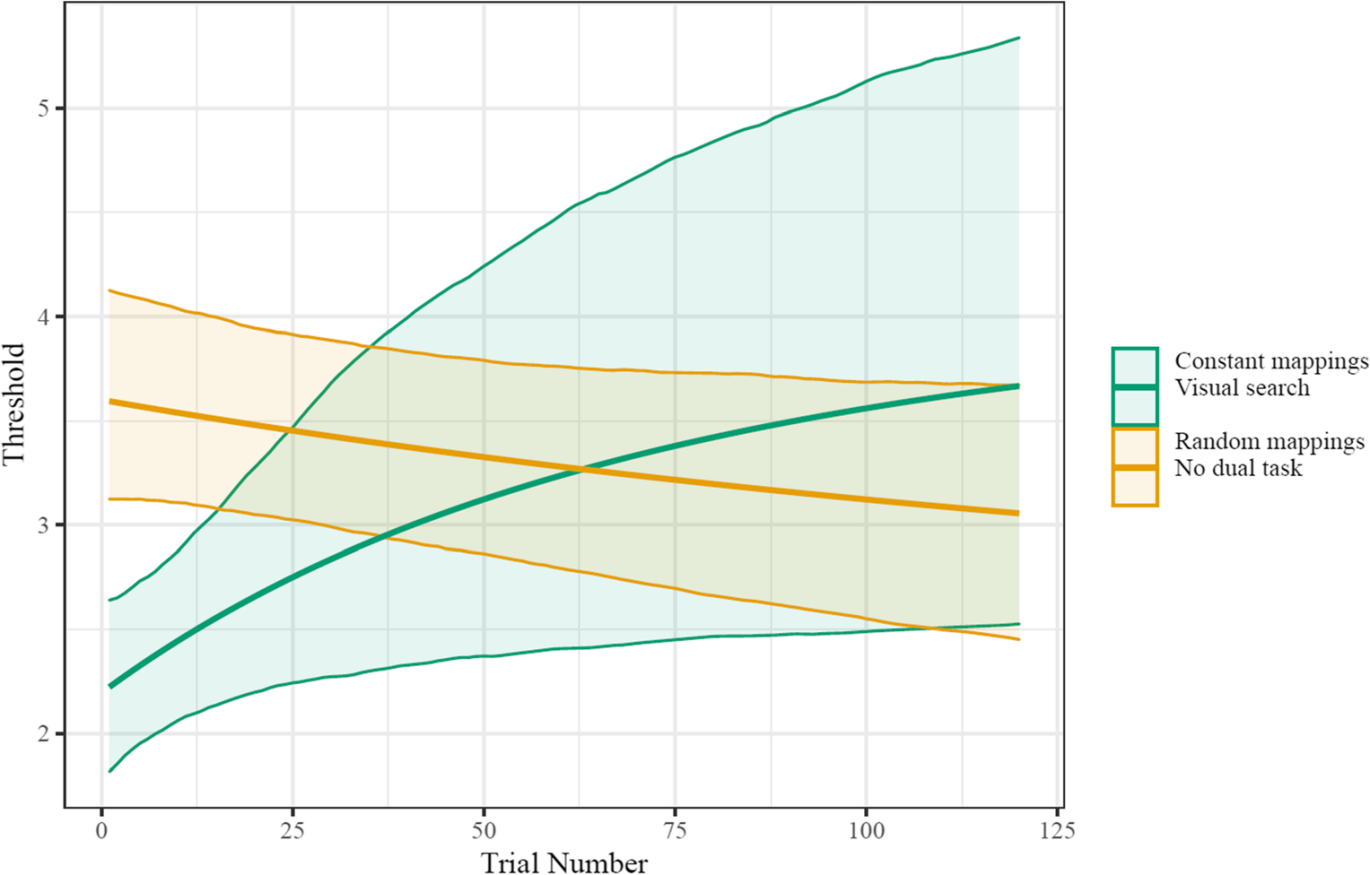
Group-level fits of change in thresholds. The condition with constant mappings and an intervening visual search task showed learning, while the random-mappings condition did not. Shaded area indicates the 95% CI of model fit values for each set size. For a visualization of the change in psychometric function as well as set sizes' accuracies over time, see the Supplementary Information (Figs. S1, S3, S5)

We found that the initial thresholds were lower [worse] for the constant-mappings with visual search condition (*n* = 31) than the constant-mappings no-dual-task condition (*b* = -0.361 CI_95_ = [-0.591,-0.148]) and the random-mappings dual-task condition (*b* = −0.483, CI_95_ = [−0.713, −0.260]; see Fig. 3), indicating that our dual task did interrupt primary-task recall. However, by the end of the task, there was no reliable difference between the visual search condition and the constant-mappings no-visual-search condition (*b* = −0.352 CI_95_ = [−1.005, 0.277], and there was no difference in the magnitude of change between the two conditions (*b* = .024, CI_95_ = [−.703, .707]). In contrast, while there was no reliable difference between asymptotic threshold with the random-mappings condition (*b* = 0.55, CI_95_ = [−0.041, 1.268] ), the constant-mappings dual-task condition did improve reliably more than the random-mappings condition (*b* = 1.038, CI_95_ = [0.353, 1.814]) When considering just participants in the dual-task condition, reliable learning was observed (i.e., higher asymptotic thresholds than starting thresholds; *b* = 0.593, CI_95_ = [0.087, 1.123]; see Supplemental Information Fig. S3).

#### Discussion

The combined results from Experiment 1 indicated that participants were able to learn how to represent stimuli in working memory as the actions that the stimuli would be used for. Such learning was in stark contrast to a condition in which stimulus–response mappings were not learnable and in which no improvement in performance was apparent over time. Learning was not solely attributable to the additional complexity involved in visually searching for a stimulus after the memory items were presented; in this condition participants’ thresholds actually improved by slightly more than in the constant-mappings no-visual-search condition.

## Experiment 2: Testing for shorter-term learning, and the possibility of learning-to-learn

### Experiment 2a. Does learning happen within blocks? Does it generalize between blocks? Does it accelerate?

Experiment 1 showed the utility of learning to represent actions in working memory, but this test of learning still lacked some key features. In the real world, as well as in the behavioral laboratory, people are likely to switch between tasks fairly frequently (e.g., moving from chopping to whisking to measuring while cooking, or moving from a forward memory span task to a backward memory span task in the psychology lab). Sequences of tasks thus have the potential to form a hierarchy of learnable features that may be generalized from one task to the next (Botvinick, 2012; Kattner, Cochrane, Cox, et al., 2017). In some senses Experiment 1 addressed this issue, by using different specific sets of six stimuli in each block, and the entire course of learning did reveal improvements in participants’ ability to represent the items in working memory. In Experiment 2, the need for generalization was taken one step further. Here, the stimulus–response mappings were not identical across blocks, thereby allowing for a test of both within-block learning and between-block generalization of learning. Specifically, on the beginning of each block, each participant’s mappings for that block were chosen randomly and then kept fixed throughout that block (i.e., “blocked” mappings). Notably, within-block learning had to happen within 30 trials, which is quite rapid and is more compatible with on-the-fly real-world situations as well as common experimental paradigms.

The model of learning used to analyze Experiment 2 was a model of change over time, which was nearly identical to Experiment 1, with the by-participant trajectories of change occurring within blocks rather than across the entire experiment (i.e., exponential function of within-block trial number 1 to 30; see Supplementary Information). The model in Experiment 2 additionally allowed each of the three parameters of the exponential function to change monotonically across blocks for each participant, that is, to either decrease or increase for a given participant with no constraint regarding the cross-block magnitudes of those increases or decreases (see Supplementary Information for further details). By-participant random intercepts for the three parameters of the exponential were also included (i.e., the starting threshold, amount of time taken to 50% of change, and asymptotic threshold). Learning was expected to be evidenced by within-condition starting thresholds being lower than the corresponding asymptotic thresholds.

#### Results

**Fig. 4:**
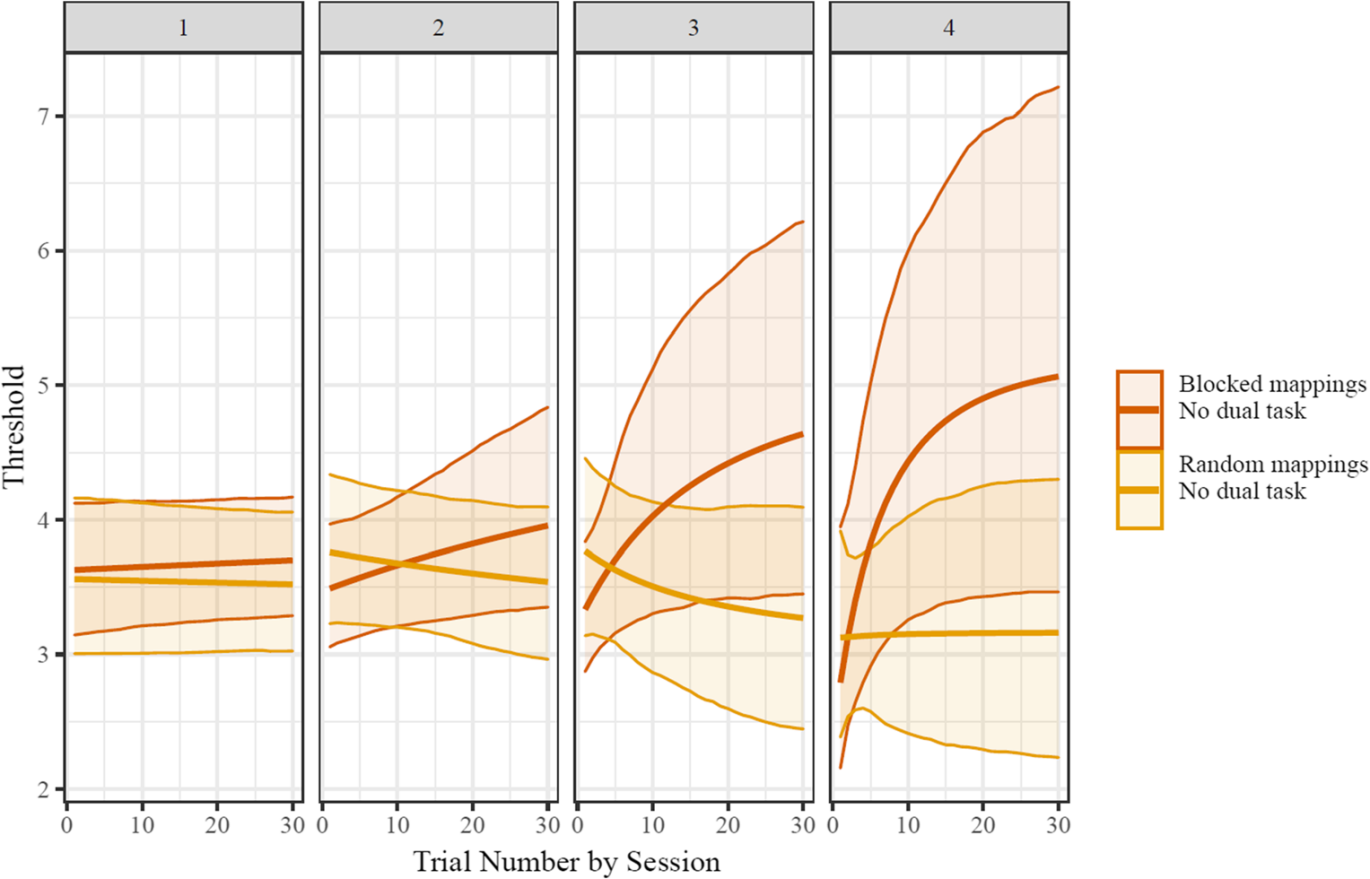
Group-level fits of within-block change in thresholds. The condition with blocked mappings showed accelerating learning, while the random-mappings condition did not. Shaded area indicates the 95% CI of model fit values for each set size. For a visualization of the change in psychometric function as well as specific set sizes’ accuracies over time, see the Supplementary Information (Figs. S2, S4, S6)

In the blocked-mapping condition with no dual task (*n* = 30), neither within-block starting thresholds (*b* = 0.091 CI_95_ = [-0.048,0.173]) nor within-block asymptotic thresholds (*b* = 0.012 CI_95_ = [−0.119, 0.249]) were reliably different between the fourth and the first blocks (in all comparisons here the fourth block is the reference, and the coefficient indicates the difference between that and the first block; see Supplemental Information Figs. S2, S4, S6). Instead, apparent improvements in performance were best explained by differences between Block 1 and Block 4 rates of change in threshold as a function of trial number (*b* = 2.312 CI_95_ = [1.753, 3.009]). That is, participants’ trajectories indicated similar eventual points of performance across all blocks when extrapolated to a large number of trials, but only in later blocks did the number of trials needed for the performance improvements decrease to a timescale that was captured by the blocks (see Fig. 4). Similar results were found by running a set of three models in which only one of the nonlinear parameters (start, rate of change, or asymptote) was allowed to change across blocks for each model. In these models the other two nonlinear parameters were still estimated using by-group fixed effects and by-participant random effects, but their values did not change from one block to the next. Using model comparison with an approximation to leave-one-out cross-validation (LOO; Vehtari et al., 2017), the model that only allowed for flexibility in rate fit much better than the models that only allowed for flexibility in starting value (ΔLOOIC = 208.5, *SE* = 35.4) or flexibility in asymptotic value (ΔLOOIC = 201.9, *SE* = 39.4). This model showed qualitatively similar results as the model allowing full flexibility (e.g., reliable differences from early to late blocks in within-block rate of change).

Similar to the blocked-mapping condition, the random-mapping condition showed no reliable changes across blocks in starting (*b* = 0.045, CI_95_ = [−0.037, 0.147]) or asymptotic (*b* = −0.126, CI_95_ = [−1.339, 0.28]) thresholds. Also like the blocked-mapping condition, in the random-mapping the timescale of change was reliably longer on Block 1 than Block 4 (*b* = 2.613, CI_95_ = [1.691, 4.061]). This result is difficult to interpret, however, since thresholds showed negligible change from start to asymptote in the random condition (*b* = 0.005, CI_95_ = [−0.46, 0.477]; see Fig. 4). Parameters in hierarchical Bayesian models are primarily informed by priors when the data does not strongly drive them; because the random-mapping condition’s fixed effects were parameterized as an offset (with a minimally informative zero-centered prior) from the constant-mapping [reference] condition (see Supplementary Information), it seems likely that its reliable block-to-block difference in rate of change is in fact spurious. This lack of improvement in performance also corresponded to somewhat lower within-block asymptotic thresholds in the random condition than the blocked condition (*b* = −0.494 CI_95_ = [−1.028, 0.013]), although this effect was not reliable. There were likewise no differences in within-block starting thresholds (*b* = 0.118 CI_95_ = [−0.434, 0.418]).

#### Discussion

Participants with sequences of within-block learnable stimulus–response mappings showed rapid learning in later blocks when compared with earlier blocks. Such a decrease in the amount of time needed to learn the stimulus–response mappings is compatible with the proposition that participants learned a hierarchical feature of the task set—namely, they learned to learn the stimulus–response mappings in order to support their effective representation of the working memory stimuli.

### Experiment 2b. Are the condition differences in Experiment 2a due to disrupted recall (e.g., visual search)?

As in Experiment 1, there remained the possibility that the contrast between the conditions reported in Experiment 2a was in fact due to the additional task demands intervening between the presentation of stimuli and the responses on the working memory task. Experiment 2b addressed this by including the same visual search task used in Experiment 1b, with all other methods remaining identical to Experiment 2a.

#### Results

**Fig. 5:**
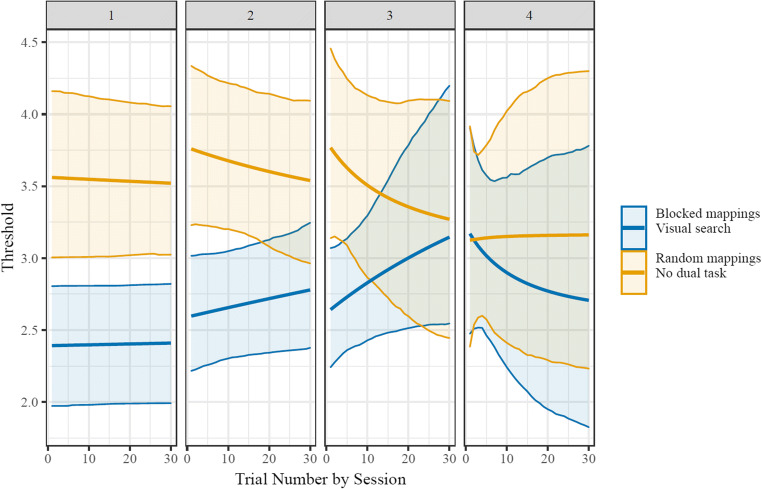
Group-level fits of within-block change in thresholds. Neither condition showed learning. Shaded area indicates the 95% CI of model fit values for each set size. For a visualization of the change in psychometric function as well as specific set sizes’ accuracies over time, see the Supplementary Information (Figs. S2, S4, S6)

In the blocked-mappings dual-task condition (*n* = 29) participants’ Block 1 starting thresholds were lower than their Block 4 starting thresholds (*b* = −0.093, CI_95_ = [−0.181, −0.003]), with the contrast between Block 1 and Block 4 asymptotic thresholds showing the opposite pattern (*b* = 0.424, CI_95_ = [0.135, 0.955]). Like the blocked-mapping condition, in the visual-search condition the timescale of change was reliably longer on Block 1 than Block 4 (*b* = 3.437, CI_95_ = [2.179, 4.917]). The within-block magnitude of change was small and variable, however, making the timescale of change difficult to interpret (*b* = −0.223, CI_95_ = [-−0.82, 0.365]; see Fig. 5). This decrement in performance corresponded to reliably lower within-block asymptotic thresholds in the visual search condition than the blocked condition with no visual search (*b* = −0.706 CI_95_ = [−1.322, −0.112]) despite there being no condition differences in within-block starting thresholds (*b* = 0.133 CI_95_ = [−0.285, 0.474]).

#### Discussion

Experiment 2b did not provide evidence that participants learned to represent working memory memoranda as action plans. Within-block learning and between-block learning-to-learn, as observed in Experiment 2a, was evidently disrupted by the inclusion of a visual search task. This disruption seems likely to have occurred due to the relative difficulty of Experiment 2b, which may have made learning extremely difficult in only 30 trials. An alternative explanation that is impossible to rule out is that the learning-to-learn in Experiment 2a was not due to participants’ increasingly representing working memory memoranda as actions but instead involved learning to more effectively remember the visual features of the stimuli themselves. That is, because this learning was prevented by the need to complete a search task prior to memory recall, it is conceivable that the increased processing load in the random-mapping condition was equivalently disrupting stimulus learning. Last, it is possible that the difficulty of completing both the memory and visual search tasks increased the extent to which participants in that condition were susceptible to fatigue-related declines toward the end of their participation; this would explain the decrease in performance during Block 4.

## General discussion

In a working memory task that did not have any overt spatial or motor-learning components, participants with predictable stimulus–response mappings were able to learn these mappings and perform better than participants with unpredictable mappings. This learning effect was not explained by a disruption in recall due to the introduction of a secondary visual search task. Further, when presented with a series of working memory tasks with consistent stimulus–response mappings within blocks but changing ones between blocks, the time necessary for participants to improve their working memory performance decreased across blocks. Such learning-to-learn effect was disrupted, however, in the presence of an intervening visual search task, indicating that the increased demands of this secondary task prevented within-block learning from being strong enough to effectively generalize to subsequent blocks.

Representations of working memory memoranda as action plans are apparently highly relevant in working memory tasks even when using standard response methods (i.e., a keyboard) rather than more complex and explicitly spatial actions (e.g., pronation or supination when rotating an input device; Gallivan et al., 2016). Clear implications thus exist for many working memory tasks which use simple and well-learned stimulus–response mappings (e.g., digit span, 2AFC judgements). Such tasks rely on relatively automatic mappings and thus allow participants to represent memoranda as upcoming actions. As a result, the ecological validity of inferences from these tasks are limited to real-world situations with highly stereotyped stimulus–response mappings. Stereotyped mappings are likely to be present in common behaviors (e.g., spreading a condiment, chopping a potato) and traditional information-centered approaches may appropriately characterize working memory in these circumstances. In contrast, other behaviors may need to be learned on-the-fly (e.g., peeling a novel fruit, turning on an oven for the first time at a friend’s house), and standard approaches to working memory may be poor models for real-world behaviors in these cases.

Participants in Experiment 2a appeared to be able to learn, not just to represent items using associated actions, but to learn a hierarchical structure facilitating successive learning. Such learning-to-learn is an exciting topic in diverse fields (Bejjanki et al., 2014; Botvinick, 2012; Gershman & Niv, 2010; Kattner, Cochrane, Cox, et al., 2017; Kruijne et al., 2021). The evidence presented here for such learning is preliminary but promising, as extensions of this learning-to-learn to effectively use working memory may bring theories explaining experimental behavior ever closer to the on-the-fly flexibility necessary for real-world behaviors. The lack of learning-to-learn in Experiment 2b indicates limits on learning-to-learn and prevents clear inferences from Experiment 2a, though, and further work is needed to probe the limits of structure learning supporting cognition.

The methods used here to identify and isolate learning in working memory performance were limited in several ways. First, while the intervening visual search task did successfully reduce performance in each Experiment, indicating some degree of interference, it was not possible to quantify whether the cost of searching in the secondary task was well-matched to the cost of searching in the memory task. Further, because verbal responses in the visual search task were not recorded and analyzed, it was not possible to assess compliance.

Second, the model-based analyses provided powerful tools for examining trajectories of performance, but they may have been overparameterized due to the sparsity of data. Especially in Experiment 2, considering several trajectories of just 30 trials each, it may have been impossible to adjudicate between differences in thresholds’ rates of change and their asymptotic levels. As indicated by our model parameter estimates as well as comparisons of reduced models, block-to-block differences in performance on a task with fairly consistent demands and stimuli seem more likely to be due to changes in rate of learning rather than change in maximum performance.

Further, given the structure of the data, it is impossible to accurately or precisely adjudicate between the gradual learning process reported in our model results and an alternative—namely, that participants explicitly noticed a pattern of consistent mappings within blocks. Such learning could in principle lead to a step-like improvement in performance once the regularity was noted, which would be able to be identified with richer sources of data than 6AFC responses (e.g., EEG decoding; van Ede et al., 2019). Step-like improvements are not apparent in the group-aggregated data (see Figs. S5 and S6), but it is possible that aggregation obscures individual-level step functions.

### Conclusion

Here we have utilized a behavioral paradigm that is superficially similar to many “span” tasks, but we have approached this task with a very different set of assumptions than more-standard approaches. Rather than the typical emphasis on the information in stimuli themselves, in which case stimulus–response mappings are likely to be well-learned already, we have shown that the process of learning the ability to represent visual stimuli as future actions goes on to support effective use of working memory. Critically, sequences of short blocks provided initial evidence for hierarchical learning and learning-to-learn to support working memory. Due to the real-world need to represent both abstract information as well as the actions relevant to it, and the need for ongoing and sequential learning in naturalistic settings, we believe that such an integrated approach to working memory allows for a more ecologically relevant investigations of working memory processes.

## Supplementary Information

ESM 1 (DOCX 878 kb)

## References

[CR1] Acheson DJ, MacDonald MC (2009). Verbal working memory and language production: Common approaches to the serial ordering of verbal information. Psychological Bulletin.

[CR2] Acheson DJ, Hamidi M, Binder JR, Postle BR (2011). A common neural substrate for language production and verbal working memory. Journal of Cognitive Neuroscience.

[CR3] Bates, D., Mächler, M., Bolker, B., & Walker, S. (2015). Fitting linear mixed-effects models using lme4. *Journal of Statistical Software, 67*(1). 10.18637/jss.v067.i01

[CR4] Bejjanki VR, Zhang R, Li R, Pouget A, Green CS, Lu Z-L, Bavelier D (2014). Action video game play facilitates the development of better perceptual templates. Proceedings of the National Academy of Sciences.

[CR5] Boettcher SEP, Gresch D, Nobre AC, van Ede F (2021). Output planning at the input stage in visual working memory. Science Advances.

[CR6] Botvinick MM (2012). Hierarchical reinforcement learning and decision making. Current Opinion in Neurobiology.

[CR7] Bürkner, P.-C. (2017). brms: An *R* Package for Bayesian multilevel models using *Stan*. *Journal of Statistical Software, 80*(1). 10.18637/jss.v080.i01

[CR8] Cochrane A (2020). TEfits: Nonlinear regression for time-evolving indices. Journal of Open Source Software.

[CR9] Cochrane A, Green CS (2021). Trajectories of performance change indicate multiple dissociable links between working memory and fluid intelligence. NPJ Science of Learning.

[CR10] Cowan N (1995). *Attention and memory*.

[CR11] D’Esposito M, Postle BR (2015). The cognitive neuroscience of working memory. Annual Review of Psychology.

[CR12] Engle RW (2002). Working memory capacity as executive attention. Current Directions in Psychological Science.

[CR13] Foster JL, Shipstead Z, Harrison TL, Hicks KL, Redick TS, Engle RW (2015). Shortened complex span tasks can reliably measure working memory capacity. Memory & Cognition.

[CR14] Fukuda K, Awh E, Vogel EK (2010). Discrete capacity limits in visual working memory. Current Opinion in Neurobiology.

[CR15] Gallivan JP, Bowman NAR, Chapman CS, Wolpert DM, Flanagan JR (2016). The sequential encoding of competing action goals involves dynamic restructuring of motor plans in working memory. Journal of Neurophysiology.

[CR16] Gershman SJ, Niv Y (2010). Learning latent structure: Carving nature at its joints. Current Opinion in Neurobiology.

[CR17] Henin S, Turk-Browne NB, Friedman D, Liu A, Dugan P, Flinker A, Doyle W, Devinsky O, Melloni L (2021). Learning hierarchical sequence representations across human cortex and hippocampus. Science Advances.

[CR18] Jaeggi SM, Buschkuehl M, Perrig WJ, Meier B (2010). The concurrent validity of the N-back task as a working memory measure. Memory.

[CR19] Kattner F, Cochrane A, Cox CR, Gorman TE, Green CS (2017). Perceptual learning generalization from sequential perceptual training as a change in learning rate. Current Biology.

[CR20] Kattner F, Cochrane A, Green CS (2017). Trial-dependent psychometric functions accounting for perceptual learning in 2-AFC discrimination tasks. Journal of Vision.

[CR21] Kruijne W, Bohte SM, Roelfsema PR, Olivers CNL (2021). Flexible working memory through selective gating and attentional tagging. Neural Computation.

[CR22] Ma WJ, Husain M, Bays PM (2014). Changing concepts of working memory. Nature Neuroscience.

[CR23] Olivers CNL, Roelfsema PR (2020). Attention for action in visual working memory. Cortex.

[CR24] Savin, C., & Triesch, J. (2014). Emergence of task-dependent representations in working memory circuits. *Frontiers in Computational Neuroscience, 8*. 10.3389/fncom.2014.00057 10.3389/fncom.2014.00057 PMC4035833 24904395

[CR25] Shipstead Z, Lindsey DRB, Marshall RL, Engle RW (2014). The mechanisms of working memory capacity: Primary memory, secondary memory, and attention control. Journal of Memory and Language.

[CR26] Trentin C, Slagter HA, Olivers CNL (2023). Visual working memory representations bias attention more when they are the target of an action plan. Cognition.

[CR27] Unsworth N, Engle RW (2007). On the division of short-term and working memory: An examination of simple and complex span and their relation to higher order abilities. Psychological Bulletin.

[CR28] van den Berg R, Shin H, Chou W-C, George R, Ma WJ (2012). Variability in encoding precision accounts for visual short-term memory limitations. Proceedings of the National Academy of Science.

[CR29] van den Berg R, Awh E, Ma WJ (2014). Factorial comparison of working memory models. Psychological Review.

[CR30] van Ede, F., & Nobre, A. C. (2022). Turning attention inside out: How working memory serves behavior. *SSRN Electronic Journal*. 10.2139/ssrn.4082572 10.1146/annurev-psych-021422-041757 35961038

[CR31] van Ede F, Chekroud SR, Stokes MG, Nobre AC (2019). Concurrent visual and motor selection during visual working memory guided action. Nature Neuroscience.

[CR32] Vehtari A, Gelman A, Gabry J (2017). Practical Bayesian model evaluation using leave-one-out cross-validation and WAIC. Statistics and Computing.

[CR33] Vogel EK, Woodman GF, Luck SJ (2001). Storage of features, conjunctions and objects in visual working memory. Journal of Experimental Psychology: Human Perception and Performance.

[CR34] Wichmann FA, Hill NJ (2001). The psychometric function: I. Fitting, sampling, and goodness of fit. Perception & Psychophysics.

[CR35] Zambrano D, Roelfsema PR, Bohte S (2021). Learning continuous-time working memory tasks with on-policy neural reinforcement learning. Neurocomputing.

[CR36] Zhang P, Zhao Y, Dosher BA, Lu Z-L (2019). Assessing the detailed time course of perceptual sensitivity change in perceptual learning. Journal of Vision.

