# Supplementary material for: Working memory is supported by learning to represent items as actions
Source: Atten Percept Psychophys. 2023 Mar 1;85(5):1649–60. doi: 10.3758/s13414-023-02654-z (PMC10372123; doi:10.3758/s13414-023-02654-z)
Supplement: Supplementary file 1 — (DOCX 878 kb) [file 13414_2023_2654_MOESM1_ESM.docx]

# Supplementary Information

## Model Specification (Experiment 1):

### TEfits code

TEbrm(

clickAcc ~ tef_link_weibull(

tef_change_expo3('totalTrialNum'

,asymForm = ~ conditionF + (stimType_c | subID)

,startForm = ~ conditionF + (stimType_c | subID)

,rateForm = ~ conditionF + (1 | subID)

)

,rhAsymptote = .1666

,threshVal = .5

,yIntercept = 1

,linkX = 'setSize')

,link_start_asym = 'exp'

,family = bernoulli(link = 'identity')

,data = .

,iter = 5000

)

Where *clickAcc* is the trial’s binary accuracy, *totalTrialNum* is the overall trial number (1:200), *trialNum* is the within-block trial number (1:30), *conditionF* is a three-level factor of conditions, *blockNumF* is a four-level ordered factor (one level for each block) with the reference being the last block, *stimType_c* is a zero-centered variable indicating which of the two stimulus types were present, *subID* was a unique code for each participant, and *setSize* was the memory load.

### Brms formula

clickAcc ~ 1 + ((0.1666 - 1) - 0.005) * (1 - 2.4775^(-(setSize/threshold)^(2.4775^shape)))

threshold ~ pAsym + ((pStart) - (pAsym)) * 2^((1 - totalTrialNum)/(2^(pRate)))

pStart ~ exp(pStartXform)

pStartXform ~ conditionF + (stimType_c | subID)

pRate ~ conditionF + (1 | subID)

pAsym ~ exp(pAsymXform)

pAsymXform ~ conditionF + (stimType_c | subID)

shape ~ conditionF + (stimType_c | subID)

## Model Specification (Experiment 2):

### TEfits code

TEbrm(

clickAcc ~ tef_link_weibull(

tef_change_expo3('trialNum'

,asymForm = ~ conditionF*mo(blockNumF) + (stimType_c | subID)

,startForm = ~ conditionF*mo(blockNumF) + (stimType_c | subID)

,rateForm = ~ conditionF*mo(blockNumF) + (1 | subID)

)

,rhAsymptote = .1666

,threshVal = .5

,yIntercept = 1

,linkX = 'setSize')

,link_start_asym = 'exp'

,family = bernoulli(link = 'identity')

,data = .

,iter = 5000

)

### Brms formula

clickAcc ~ 1 + ((0.1666 - 1) - 0.005) * (1 - 2.4775^(-(setSize/threshold)^(2.4775^shape)))

threshold ~ pAsym + ((pStart) - (pAsym)) * 2^((1 - trialNum)/(2^(pRate)))

pStart ~ exp(pStartXform)

pStartXform ~ conditionF * mo(blockNumF) + (stimType_c | subID)

pRate ~ conditionF * mo(blockNumF) + (1 | subID)

pAsym ~ exp(pAsymXform)

pAsymXform ~ conditionF * mo(blockNumF) + (stimType_c | subID)

shape ~ conditionF * mo(blockNumF) + (stimType_c | subID)

## Results of Experiment 2 models with reduced parameter flexibilities

In Experiment 2, all three parameters of within-block change (start, rate, and asymptote) were estimated as changing from one block to the next. We tested a set of 3 reduced models in which two of these parameters were not allowed to change from block to block, but were instead estimated as being the same across all blocks (for each condition and participant). In the model code above this corresponds to the removal of the mo(blockNumF) term from all parameter formulas except one. The results of model comparisons indicated that the model that retained flexibility for the rate parameter fit much better than the models that allowed flexibility for starting or asymptotic thresholds.

## Psychometric function change (Experiment 1):


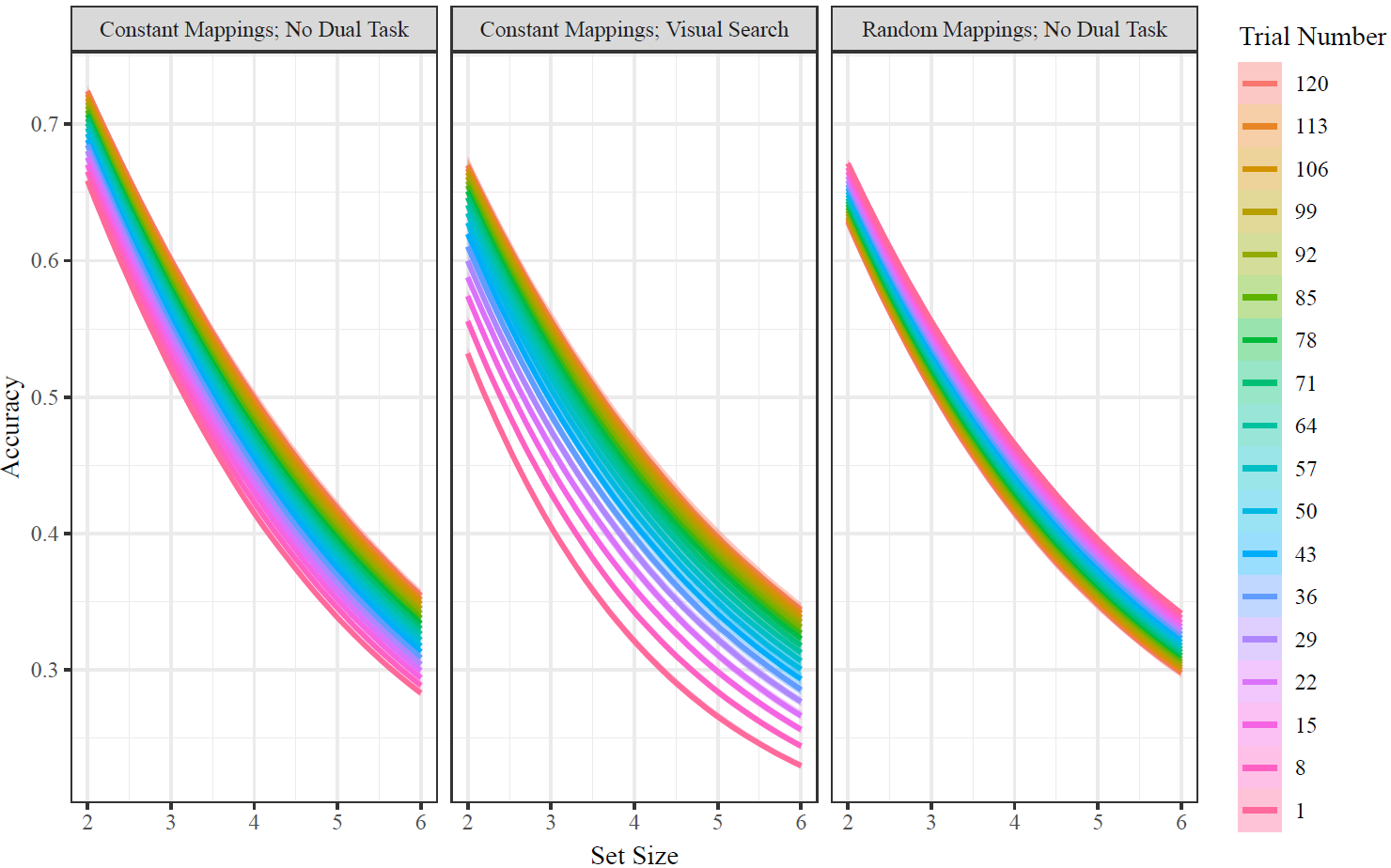


*Figure S1. Group-level estimates of changes in psychometric functions in Experiment 1 for each condition (column). Psychometric functions were evaluated on every 7^th^ trial for demonstration purposes.*

## Psychometric Function change (Experiment 2):


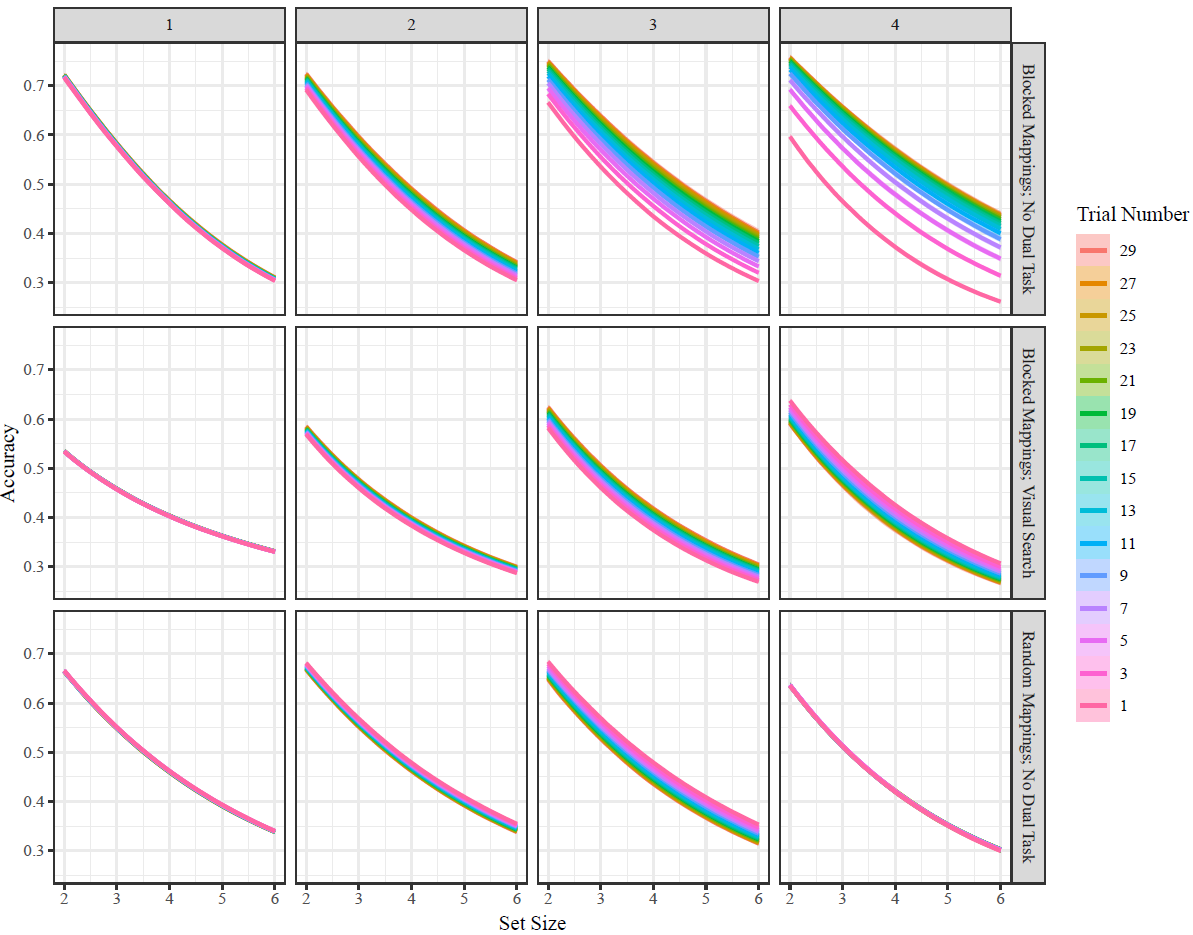


*Figure S2. Group-level estimates of changes in psychometric functions in Experiment 2 for each condition (row) and block (column). Psychometric functions were evaluated on every 2^nd^ trial for demonstration purposes.*

## Psychometric Function compared to average data (Experiment 1)


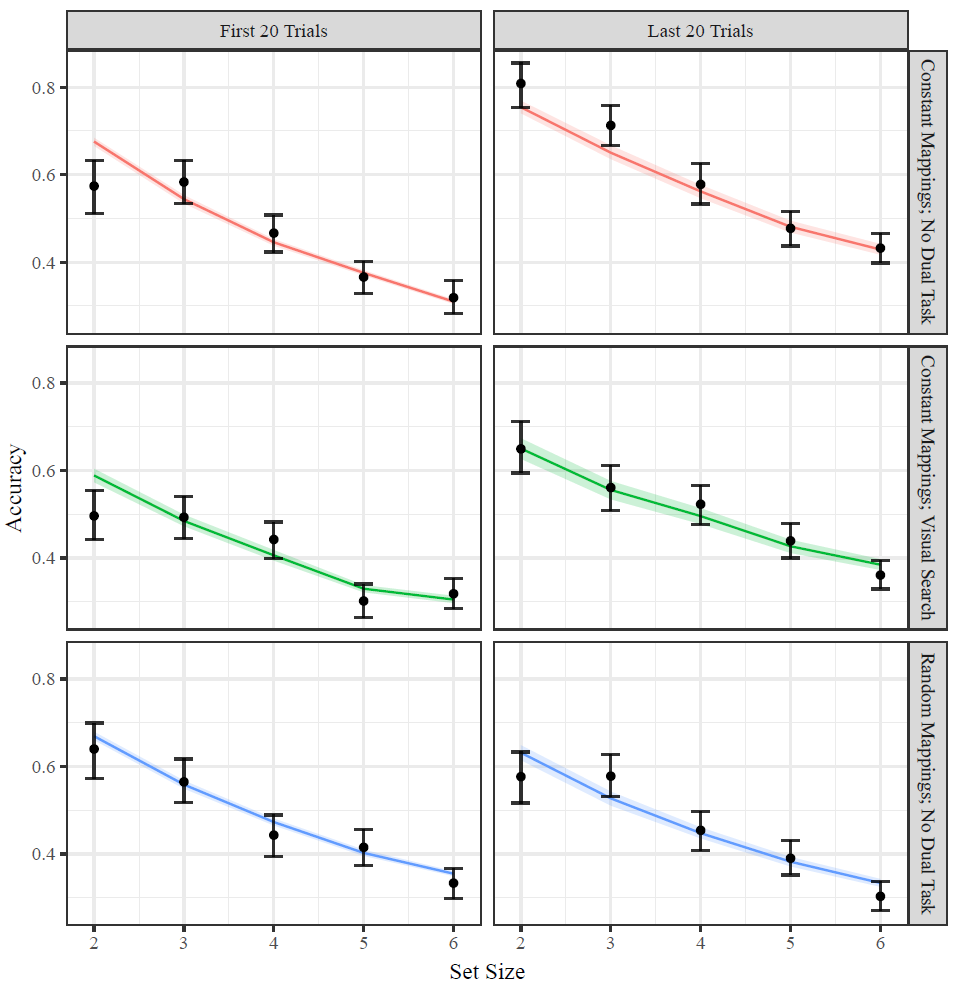


*Figure S3.* *Fitted psychometric function (colored lines) and raw accuracy (black dots) averaged for the first and last 20 trials of Experiment 1. Error bars and shaded area indicate bootstrapped 95% CI.*

## Psychometric Function compared to average data (Experiment 2)


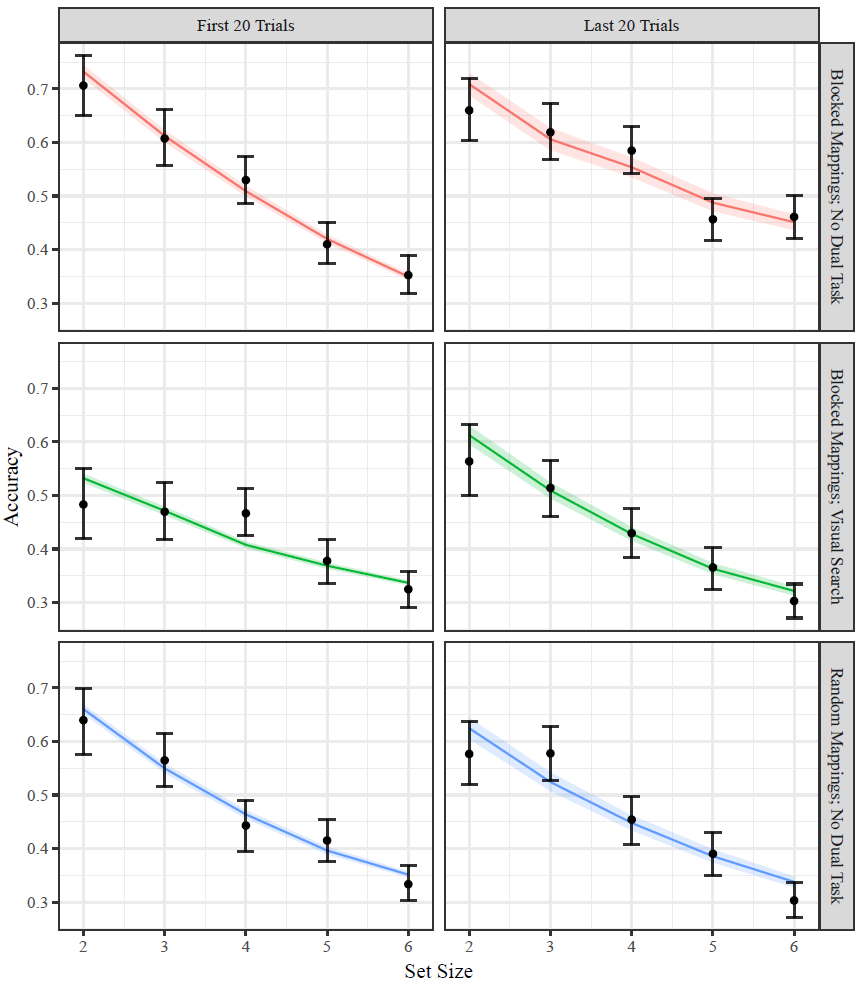


*Figure S4. Fitted psychometric function (colored lines) and raw accuracy (black dots) averaged for the first and last 20 trials of Experiment 2. Error bars and shaded area indicate bootstrapped 95% CI.*

## Model fit values compared to running average of data (Experiment 1)


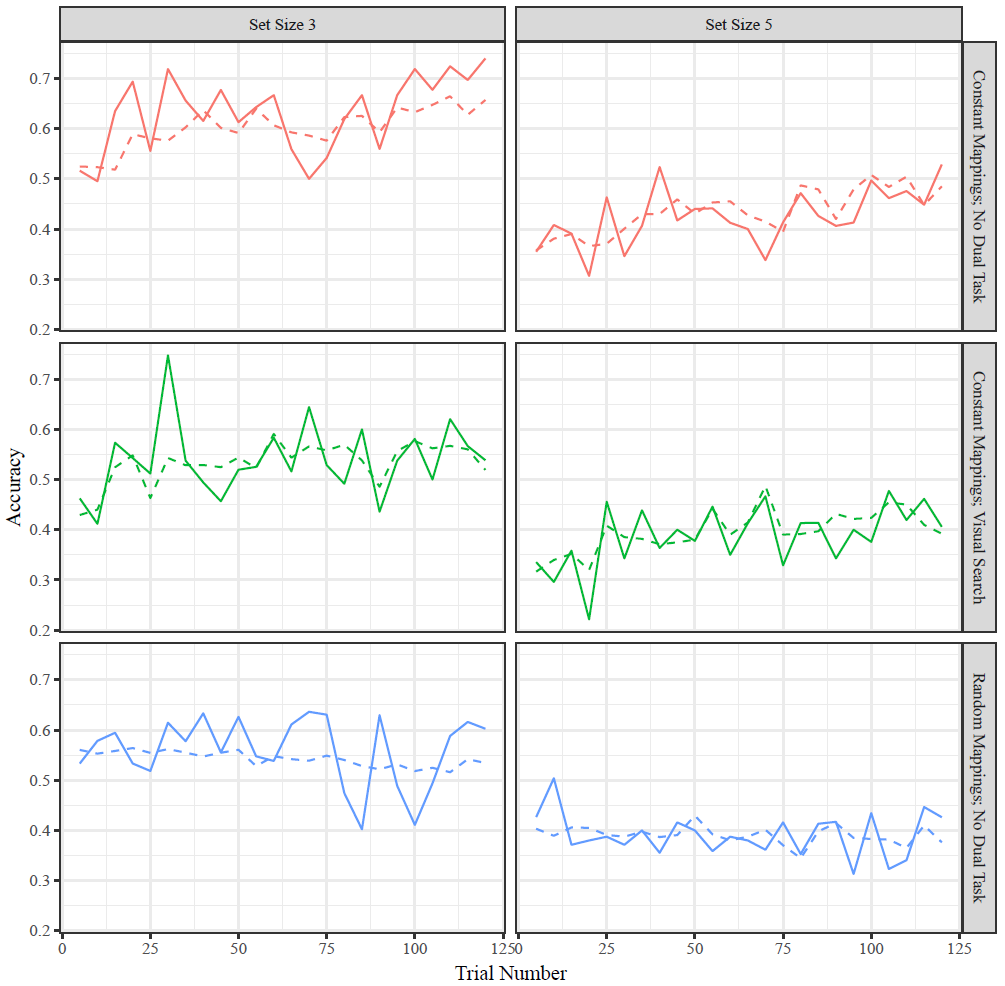


*Figure S5. Data was separated into bins of 5 trials, for each of which average participant accuracy (solid line) and model fit accuracy (dashed line) are plotted. Data is separated by group (rows). Each column shows one set size because averaging accuracies across set sizes obscures the pattern of results and model fits. Set sizes 3 and 5 were chosen to plot for demonstration purposes. Guessing rate would be .1667. Note that much of the variation from one bin to the next is due to stimulus randomization, with some bins including more trials of that set size from participants who tended to performed worse and other bins including more trials of that set size from participants who tended to perform better.*

## Model fit values compared to averages of data (Experiment 2)


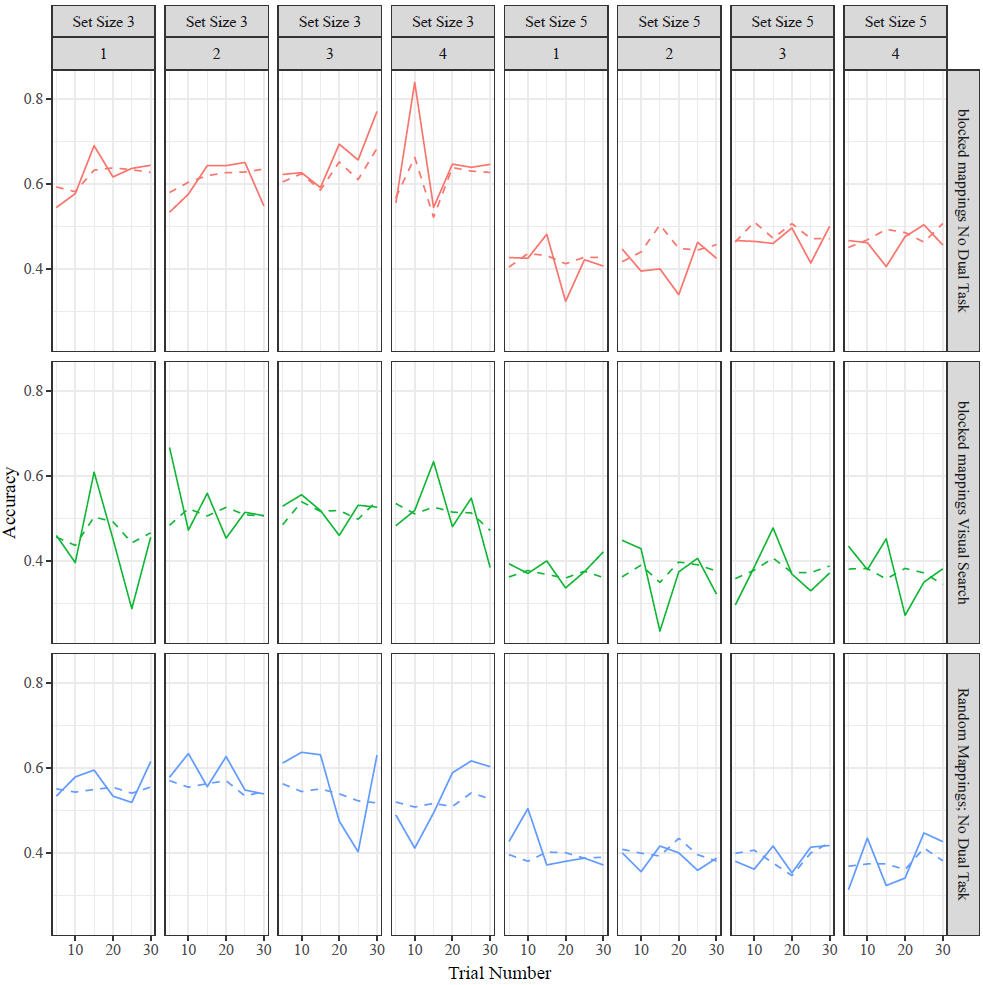


*Figure S6. Data was separated into bins of 5 trials, for each of which average participant accuracy (solid line) and model fit accuracy (dashed line) are plotted. Data is separated by group (rows). Each column shows one set size (top label) because averaging accuracies across set sizes obscures the pattern of results and model fits. Set sizes 3 and 5 were chosen to plot for demonstration purposes. Guessing rate would be .1667. Columns are also divided by block (bottom label shows block number). Note that much of the variation from one bin to the next is due to stimulus randomization, with some bins including more trials of that set size from participants who tended to performed worse and other bins including more trials of that set size from participants who tended to perform better.*

|  | **Control Group.** | **Experiment 1a.** | **Experiment 1b.** | **Experiment 2a.** | **Experiment 2b.** |
| --- | --- | --- | --- | --- | --- |
| **Participant N** | 30 | 31 | 31 | 30 | 29 |

*Table S1. Participant n per condition.*
